# The impact of the rotavirus vaccine on diarrhoea, five years following national introduction in Fiji

**DOI:** 10.1016/j.lanwpc.2020.100053

**Published:** 2020-11-25

**Authors:** Adam W.J. Jenney, Rita Reyburn, Felisita T. Ratu, Evelyn Tuivaga, Cattram Nguyen, Sokoveti Covea, Sarah Thomas, Eric Rafai, Rachel Devi, Kathryn Bright, Kylie Jenkins, Beth Temple, Lisi Tikoduadua, Joe Kado, E. Kim Mulholland, Carl D. Kirkwood, Kimberley K. Fox, Julie E. Bines, Varja Grabovac, Aalisha Sahu Khan, Mike Kama, Fiona M. Russell

**Affiliations:** a Infection and Immunity, Murdoch Children's Research Institute, Parkville, VIC, Australia; b College of Medicine, Nursing and Health Sciences, Fiji National University, Suva, Fiji; c Ministry of Health and Medical Services, Suva, Fiji; d Australia's Support to the Fiji Health Sector, Suva, Fiji; e Division of Global and Tropical Health, Menzies School of Health Research, Darwin, NT, Australia; f Department of Infectious Disease Epidemiology, London School of Hygiene & Tropical Medicine, London, UK; g Enteric and Diarrheal Diseases, Global Health, Bill and Melinda Gates Foundation, Seattle, USA; h Regional Office for the Western Pacific, World Health Organization, Manila, Philippines; i Department of Gastroenterology and Clinical Nutrition, Royal Children's Hospital, Melbourne, VIC, Australia; j Department of Paediatrics, The University of Melbourne, Melbourne, VIC, Australia; k Division of Pacific Technical Support, World Health Organization, Suva, Fiji; l Centre for International Child Health, Department of Paediatrics, The University of Melbourne, Parkville, VIC, Australia

## Abstract

**Background:**

In 2012, Fiji became the first independent Pacific island country to introduce rotavirus vaccine. We describe the impact of rotavirus vaccine on all-cause diarrhoea admissions in all ages, and rotavirus diarrhoea in children <5 years of age.

**Methods:**

An observational study was conducted retrospectively on all admissions to the public tertiary hospitals in Fiji (2007–2018) and prospectively on all rotavirus-positive diarrhoea admissions in children <5 years at two hospital sites (2006–2018, and 2010–2015), along with rotavirus diarrhoea outpatient presentations at one secondary public hospital (2010–2015). The impact of rotavirus vaccine was determined using incidence rate ratios (IRR) of all-cause diarrhoea admissions and rotavirus diarrhoea, comparing the pre-vaccine and post-vaccine periods. All-cause admissions were used as a control. Multiple imputation was used to impute missing stool samples.

**Findings:**

All-cause diarrhoea admissions declined among all age groups except among infants ≤2 months old and adults ≥55 years. For children <5 years, all-cause diarrhoea admissions declined by 39% (IRR)=0•61, 95%CI; 0•57–0•65, *p*-value<0•001). There was an 81% (95%CI; 51–94%) reduction in mortality among all-cause diarrhoea admissions in children under <5 years. Rotavirus diarrhoea admissions at the largest hospital among children <5 years declined by 87% (IRR=0•13, 95%CI; 0•10–0•17, *p*-value<0•001). Among rotavirus diarrhoea outpatient presentations, the IRR was 0•39 (95%CI; 0•11, 1.21, *p*-value=0.077).

**Interpretations:**

Morbidity and mortality due to rotavirus and all-cause diarrhoea in Fiji has declined in people aged 2 months to 54 years after the introduction of the RV vaccine.

**Funding:**

Supported by WHO and the Australian Government.

## 1 Introduction

Beyond the neonatal period, diarrhoea is the second commonest cause of death in children less than five years old, worldwide [1]. Without vaccination, rotavirus causes approximately 38% of all diarrhoea admissions in children <5 years old [2]. Two vaccines became available in 2006 [3] and yet in 2015 there were still 199,000 diarrhoeal deaths in children due to rotavirus [4]. Approximately 45% of infants do not have access to the vaccine [5] and so rotavirus remains common [6]. However, where the vaccine has been introduced into low- and middle-income countries (LMIC), the Global Rotavirus Surveillance Network has reported a 39•6% reduction in diarrhoea admissions [2]. Rotavirus vaccine impact is also evident among outpatients, where most of the cases are seen [7].

Island nations in the Pacific may particularly benefit from the rotavirus vaccine due to the risk of severe rotavirus outbreaks [8,9]. However, with the exception of Fiji and the Philippines, the burden of rotavirus in the Asia-Pacific region is largely unknown [10], [11], [12]. In October 2012, Fiji was the first independent Pacific Island country to introduce rotavirus vaccine into the national immunisation schedule. This decision was based on our previous findings that 39% of diarrhoea admissions in children <5 years of age, particularly indigenous (iTaukei) Fijian children, prior to vaccine introduction were due to rotavirus [10]. In order to inform future policy decisions in Fiji and other Pacific islands we undertook a study to describe rotavirus diarrhoea inpatient and outpatient attendances before and after the national introduction of rotavirus vaccine in children; and all-cause diarrhoea admission rates, in all ages, nationwide.

## 2 Methods

### 2.1 Study setting and design

Fiji is an upper-middle income Pacific island country of 884,887 (2017 census) of whom 55•9% reside in an urban setting [13]. The ethnic distribution of residents in Fiji for 2017 was 62% iTaukei, 32% Fijian of Indian descent and 6% other, estimated from the 2007 census data with ethnicity specific growth rates applied [14]. Women comprise 49% of the population [14]. In 2017, the post neonatal infant mortality rate in Fiji was 11 per 1000 live births [15]. Malnutrition rates among children <5 years were 7.5%, 6.3% and 5.1% for stunting, wasting and overweight respectively when last published in 2004 [16].

In October 2012, the Government of Fiji introduced into the national immunisation schedule; the monovalent rotavirus vaccine (Rotarix, GlaxoSmithKline) as a two-dose schedule at six and 14 weeks of age and the 10-valent pneumococcal conjugate vaccine (Synflorix, GlaxoSmithKline) as a three-dose schedule at six, 10 and 14 weeks of age. National vaccine coverage estimates for two doses of rotavirus vaccine were 85%, 91%, 94%, 93% and 98% in 2013, 2014, 2015, 2016 and 2017, respectively [17].

The percentage of all admissions in Fiji between 2007 and 2017 to the three tertiary hospitals were; 37% Colonial War Memorial Hospital (CWMH), Suva, 19% Lautoka, 11% Labasa, and 33% were to secondary hospitals. Upon admission to a tertiary hospital, patients’ details are recorded into the ward registers by a nurse. Doctors manually record clinical notes, including discharge diagnoses. Coders assign an ICD-10-AM code for each discharge diagnosis, which are entered, with the other clinical information into the computerised national hospital admission database (PATIS) which generates a patient's unique National Health Number (NHN). The PATIS system has been available at all three tertiary hospitals from 2007 and has been demonstrated in a previous study to be 89% complete [18].

This is an observational study describing all-cause diarrhoea admissions nationwide in all ages at all three public tertiary hospitals (CWMH, Lautoka, Labasa) and rotavirus diarrhoea in <5-year olds at two hospital-based sentinel sites: the largest tertiary hospital for admissions CWMH, and one secondary public hospital for admissions and outpatients, Savusavu Hospital.

### 2.2 National all-cause diarrhoea admission data

Hospital admission data for all-causes of illness were retrospectively extracted from PATIS for people of all ages between 2007 and 2017. All-cause diarrhoea admissions and all-cause admissions (used as a control group to assess if changes in the post-rotavirus vaccine period were due to changes in access to care) were classified according to primary discharge diagnosis using ICD-10-AM codes; all-cause diarrhoea were ICD-10-AM codes: A0-A2.1, A2.3-A6.3, A6.9-A9, A9.9, K52.9. All-cause admissions were all ICD-10-AM codes excluding those relating to diarrhoea (A0-A2.1, A2.3-A6.3, A6.9-A9, A9.9, K52.9), pneumonia (all ‘J’ codes – as the pneumococcal conjugate vaccine was introduced simultaneously with the rotavirus vaccine and reductions in pneumonia admissions have been found), child birth (all ‘O’ codes) and mental health (all ‘F’ codes). Readmissions for any cause within 30 days were identified by NHN and excluded as the second admission was considered the same episode of illness. Any child discharged with diarrhoea representing with subsequent malnutrition (or any other condition that did not have the ICD10 code for diarrhoea, pneumonia and pneumococcal related disease) on day 31 or after, would be counted as a case, and subsequently as a control condition.

### 2.3 Sentinel site surveillance

There were two rotavirus diarrhoea surveillance sites: CWMH, serving an estimated population of 34,920 children <5 years of age (38% of Fiji's total) [13] in 2017; and Savusavu Hospital serving 6563 semi-urban and rural children <5 years of age (7% of the national total) [13]. Population data was sourced from the Fiji Bureau of Statistics 2007 census as the 2017 census did not record ethnicity and our time period of analysis started in 2006. The population data was adjusted for population growth by multiplying the age and ethnic specific population data from the previous year by the ethnic specific annual growth rate; +1.7% for iTaukei, −1.7% for Fijians of Indian descent and +0.7% for others [14].

To obtain data on rotavirus positive outcomes, a diarrhoea prospective surveillance system was established in December 2005 at CWMH. Cases were identified daily (by our research staff Monday-to-Friday and ward nursing staff on weekends), through checking paediatric ward registers and PATIS to ensure all cases were included. Identifiers were checked and any duplicates were removed. Children <5 years of age admitted to the CWMH with non-bloody diarrhoea (defined as 3 or more looser than normal stools [without blood] within 24 h of admission, based on parental report) were enroled. Demographic and clinical data were recorded; age, ethnicity, sex, length of stay in hospital, treatment, death in hospital. Nurses or parents collected stool samples within 48 h of admission. Ethnicity was self-reported in surveillance and PATIS. For the surveillance at CWMH between December 2005 to June 2012 all enrolment procedures were done by study staff. In June 2012 Ministry of Health and Medical Services (MoHMS) collected the data. The nurse-in-charge oversaw enrolment and study staff monitored stool and data collection daily (Monday-to-Friday). Stool samples were stored (4–8 °C) prior to transportation to the Fiji Centre for Communicable Disease Control in Suva (15 min drive from CWMH).

Similarly, at Savusavu Hospital, children (both inpatients and outpatients) were enroled between March 2010 and December 2016. Cases were identified from admission notes and ward registration (as PATIS is unavailable in Savusavu). A study-appointed technical assistant oversaw the surveillance and undertook; data collection, stool collection and storage. Stool samples were stored at −20 °C and transported to the Fiji Centre for Communicable Disease Control in Suva using ice packs every three months.

At the Fiji Centre for Communicable Disease Control in Suva stool samples were stored at −70 °C or −20 °C depending on the availability of space and tested in batches on a three-monthly basis. Rotavirus antigen was detected using the ProSpecT Rotavirus microplate assay (Oxoid, Basingstoke, Hampshire, UK). Specimens were sent on dry ice to the regional reference laboratory (RRL), Murdoch Children's Research Institute, Melbourne on a six-monthly basis, where real-time multiplex PCR was used to confirm the presence of rotavirus and determine the virus genotype [19]. Genotyping results will be presented elsewhere.

### 2.4 Surveillance monitoring

At both sites monitoring was conducted by a study staff, on a daily (Monday to Friday) basis at CWMH and a quarterly basis at Savusavu. A diarrhoea case identified post-discharge, was enroled retrospectively noting the absence of stool collection.

### 2.5 Data analysis

The years defining the pre-rotavirus vaccination period are displayed in Table 1, with justification for years selected in the supplementary material. Full calendar years were used due to the seasonality, except for Savusavu where all available pre-rotavirus vaccine data was used (as the study period there was relatively short).

**Table 1:** Years included in the pre- and post-rotavirus vaccination period by site and outcome.

Outcome	Site	Pre-vaccination period	Post-vaccination period
All-cause diarrhoea admissions	National	January 2007 to December 2011	January 2014 to December 2017
All-cause diarrhoea admissions	CWMH	January 2006 to December 2011	January 2014 to December 2018
RV diarrhoea admissions	CWMH	January 2006 to December 2011	January 2014 to December 2018
RV diarrhoea admissions	Savusavu	May 2010-December 2011	January 2014 to December 2015
RV diarrhoea outpatient presentations	Savusavu	May 2010-December 2011	January 2014 to December 2015

RV=rotavirus.

CWMH=Colonial War Memorial Hospital.

Age-stratified analyses were planned *a priori* (e.g. to allow comparisons across studies, or to enable assessment of incidence rates in vaccinated and unvaccinated age groups). Age groups were; infants too young to be vaccinated ≤2 months of age, infants fully vaccinated 1–11 months of age, <1 year of age, 1–2 years of age to determine waning in the second year of life, 2–4 years of age, <5 years of age, 5–9 years of age (unvaccinated and most likely to benefit for indirect effects in the household), 10–19 years of age (preteen and adolescents), 20–54 years of age (caregivers) and ≥55 years of age (elderly in this population).

Demographic characteristics were compared between those with and without stool specimens using a chi squared test of association for binary data and Wilcoxon rank-sum for continuous data.

Incidence rates were calculated as the number of cases, divided by the population, multiplied by 100,000 with exact Poisson confidence intervals. The incidence rate ratio post/pre-rotavirus vaccine was calculated using Poisson regression with rotavirus counts as the outcome variable and period (pre-vaccine vs. post-vaccine) as a covariate: thus providing an overall estimate of vaccine impact. The logarithm of the population denominators was included in the model as an offset variable. The model coefficients were exponentiated to obtain incidence rate ratios (with corresponding 95% confidence intervals and *p*-values). We considered subgroup analysis by ethnicity to examine whether declines in incidence rates were different between ethnic groups. To determine whether to present ethnicity-stratified analyses, we included terms for the interaction between ethnicity and vaccine period in our models. To do this we fitted Poisson regression models with counts of rotavirus positive cases as the outcome variable, and vaccine period (baseline vs. post-vaccination), ethnicity and an interaction term as covariates, and used a *p*-value of 0.05 as the significance level for interactions. When investigating interactions, ethnicity was treated as a binary variable (iTaukei vs. Fijian of Indian descent); the “other” group was omitted due to small numbers and the diversity within this group. The outcome ‘all-cause admissions’ was analysed using the same method and used as a control to assess changes in hospital admissions during the period of analysis.

We performed two sensitivity analyses; the first, to adjust for cases without a stool sample collected, was multiple imputation to handle missing data for a rotavirus test result. The second adjusted for changes in trends over the years not associated with the vaccine, i.e. an interrupted time series regression analysis of national all-cause diarrhoea admissions in children <5 years, the only outcome among young children with enough data points for time-series analysis. Full methods of both sensitivity analyses are described in the supplementary material.

The case fatality rates (CFR) were calculated for children aged 0–59 months of age admitted to CWMH with all-cause diarrhoea, as the number of deaths in hospital among children admitted with all-cause diarrhoea divided by the number of children admitted for all-cause diarrhoea multiplied by 100, with exact Poisson confidence intervals. All data were analysed using STATA 15•1 (StataCorp LP, Texas, USA).

The manuscript has been prepared according to the STROBE (STrengthening the Reporting of OBservational studies in Epidemiology) checklist.

### 2.6 Ethics approval

Ethical approval for these studies was obtained from the Fiji National Research Ethics Review Committee (number 2013•40) and from the University of Melbourne Human Research Ethics Committee for the initial study surveillance in CWMH 2005–2012 (Ethics ID: 050546X) and Savusavu from 2010 to 2012 (Ethics ID: 0931282), during this period written informed consent was obtained from the participants’ parents. From June 2012, the MoHMS considered this public health surveillance no longer required written consent. Retrospective data extracted from PATIS was deidentified.

### 2.7 Role of the funding source

This work was supported by the World Health Organisation to set up the initial study (2005–7) and an additional World Health Organisation grant [Registry File No. V27-181-188] for data collected between 2006 and 2013. A Merck investigator-initiated grant (IISP ID#: 35,248) funded the early work in Savusavu (2009–10). Data collection between 2014 and 2018 and the final analysis was supported by the Department of Foreign Affairs and Trade of the Australian Government and Fiji Health Sector Support Program (FHSSP). FHSSP is implemented by Abt JTA on behalf of the Australian Government. Funders had no role in study design, data collection, data analysis, interpretation, writing of the report. RR had full access to study data and AWJJ had final responsibility for the decision to submit for publication.

## 3 Results

### 3.1 Description of data and demographics

There were 8851 all-cause diarrhoea admissions nationally for all ages between 2007 and 2017 (Table 2). National all-cause diarrhoea admissions were more common among the iTaukei (6279, 71%) population vs. Fijians of Indian descent (2164, 25%), and less common among males (3886, 44%) than females as compared to the population distribution as described in the methods (Table 2).

**Table 2:** Demographics and clinical characteristics of all-cause diarrhoea admissions and outpatient presentations in all ages, Fiji.

Characteristics	National all-cause diarrhoea admissions^a^ 2007–2017		CWMH all-cause diarrhoea admissions 2007–2018	Savusavu all-cause diarrhoea admissions May 2010-Dec 2015	Savusavu all-cause diarrhoea outpatient presentations May 2010-Dec 2015
	All ages	Children <5yrs	Children <5yrs		
	***n*** **=** **8851**	***n*** **=** **4353**	***n*** **=** **3312**	***n*** **=** **179**	***n*** **=** **1260**
*Age, median (IQR)*	*n* = 8851	*n* = 4353	*n* = 3311	*n* = 179	*n* = 1259
	5yrs (1–30yrs)	16 m (9–29 m)	13 m (7–23 m)	17 m (11–29 m)	19 m (11–30 m)
*Ethnicity, n (%)*	*n* = 8851	*n* = 4353	*n* = 2982	*n* = 89	*n* = 498
i-Taukei	6279 (70•9)	3284 (75•4)	2347 (78•7)	79 (88•8)	335 (67•3)
Fijians of Indian descent	2164 (24•5)	836 (19•2)	447 (15•0)	5 (5•6)	117 (23•5)
Others	408 (4•6)	233 (5•4)	188 (6•3)	5 (5•6)	46 (9•2)
*Male, n (%)*	*n* = 8851	*n* = 4353	*n* = 3311	*n* = 179	*n* = 1260
	3886 (43•9)	2562 (58•9)	1967 (59•4)	101 (56•4)	714 (56•7)
*Length of stay in days, median (IQR)*	*n* = 8832	*n* = 4353	*n* = 3180	*n* = 75	
	3 (2–6)	2 (1–4)	2 (1–4)	3 (1–4)	–
*Days with diarrhoea before admission, median (IQR)*			*n* = 2583	*n* = 76	*n* = 180
	–	–	2 (1–3)	2 (1–4)	2 (1–3)
*Received IV fluid, n (%)*			*n* = 2685	*n* = 161	*n* = 1048
	–	–	1663 (61•9)	94 (58•4)	16 (1•5)
*Vomiting, n (%)*			*n* = 1425	*n* = 81	*n* = 349
	–	–	1127 (79•1)	52 (64•2)	156 (44•7)
*Died during admission, n (%)*	*n* = 8828	*n* = 4352	*n* = 3206	*n* = 174	*n* = 1225
	133 (1•5)	33 (0•8)	61 (1•9)	0	3 (0•2)

a ICD codes A0-A2•1, A2•3-A6•3, A6•9-A9, A9•9, K52•9. Data regarding the variables ‘days with diarrhoea before admission’, ‘received IV fluid’ and ‘vomiting’ were not collected for the national all-cause diarrhoea admissions as this is not recorded in PATIS.

Between 2007–2017, 71% (3087/4353) of nationwide all-cause diarrhoea admissions among children <5 years of age occurred at CWMH. Stool sample testing rates in the pre- vs. post-rotavirus vaccine period were 80% (1498/1854) vs. 57% 9586/1037) *p*-value<0.001; 52% (44/84) vs. 97% (38/39) *p*-value <0.001; and 23% (79/347) vs. 83% (432/520) *p*-value<0.001, at CWMH, Savusavu inpatients and outpatients, respectively (Supplementary Table 1). Among CWMH cases 60% (3/5) of the demographic or clinical characteristics differed between those with and without stool sample collected; the former were slightly older; 14 months (interquartile range (IQR); 8–24 m) vs. 12 months (IQR; 7–23 m) *p*-value=0.056; had a longer periods of admission; two days (IQR; 1–40 days) vs. two days (IQR; 1–4 days) *p*-value<0.001; were less likely to have received intravenous fluid; 417 (53.4%) vs. 1246 (65.4%) *p*-value<0.001. Rotavirus positivity rates at CMWH using the raw data were 39.9% (599/1498) and 12.3% (72/586) in the pre- and post-rotavirus vaccine periods, respectively and using the multiple imputation data were 39.2% (95%CI: 1.3 to 36.7) and 11.8% (95%CI: 9.2 to 14.4%) in the pre- and post-rotavirus vaccine periods, respectively.

### 3.2 Vaccine impact on national all-cause diarrhoea admissions

**Fig. 1:**
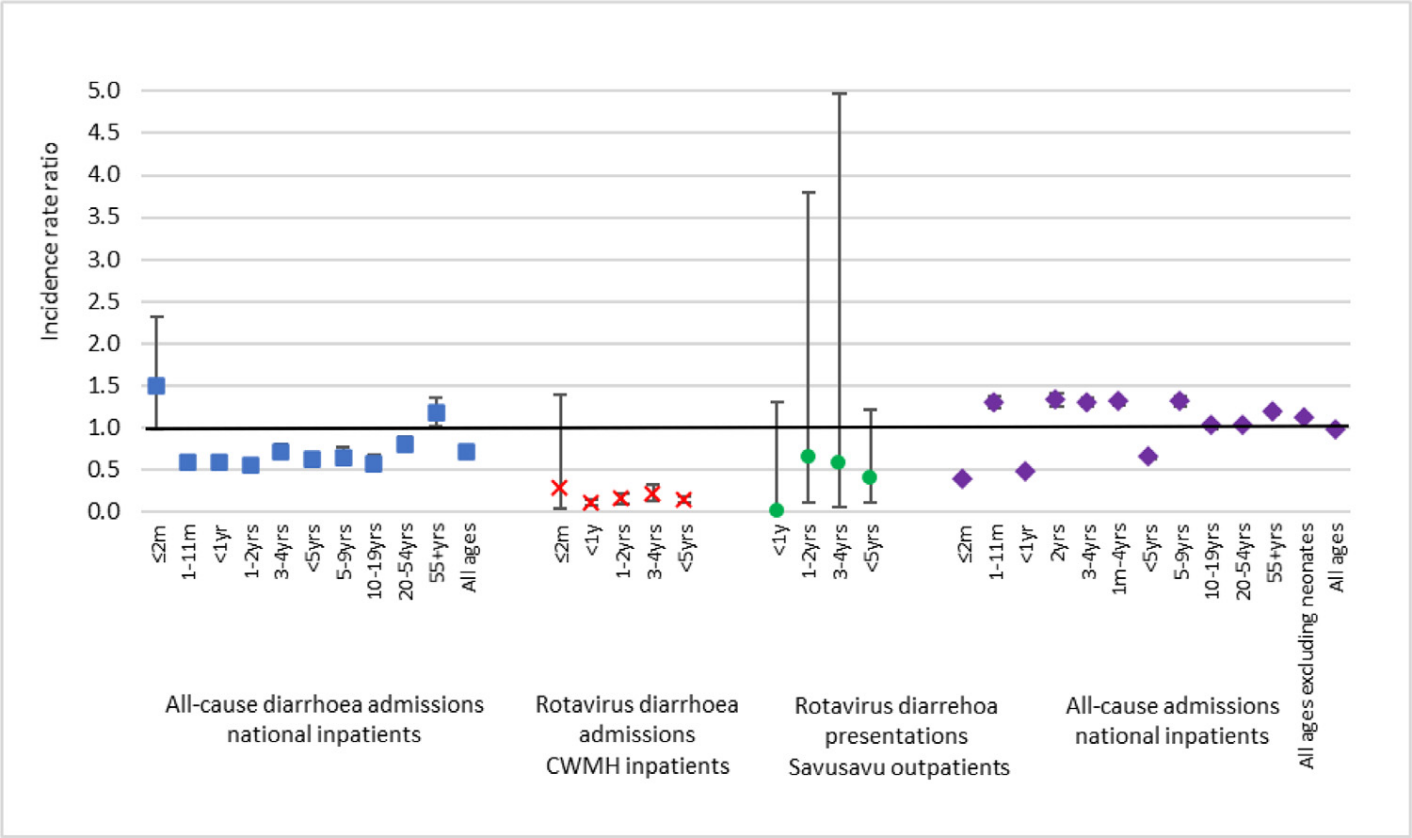
Incidence rate ratios (post-rotavirus vaccine vs. pre-rotavirus vaccine introduction) for all-cause diarrhoea and all cause admissions and rotavirus diarrhoea CWMH admissions, Savusavu inpatients and Savusavu outpatients. An incidence rate ratio above one (the black line) represents an increase in incidence rate from the pre-rotavirus vaccine period and an incidence rate ratio below one represents a decrease in incidence rate from the pre-rotavirus vaccine period.

All-cause diarrhoea admissions declined in the post-rotavirus vaccine period among all age groups except infants ≤2 months old and adults ≥55 years, where it increased (Table 3, Fig. 1 and Supplementary Table 2). The time-series analysis suggests a stable background trend in diarrhoea over time (IRR=1.00 (1.00, 1.01) for the monthly change in diarrhoea rates), and an estimated 52.3% (95% CI: 27.7% to 68.5%) decrease in monthly all-cause diarrhoea admissions following vaccine introduction (Supplementary Table 3 and Supplementary Fig. 1). Full results of are presented in the supplementary materials.

**Table 3:** Mean annual cases and incidence rates in the pre- and post-rotavirus vaccine periods in Fiji, and incidence rate ratios estimating post/pre-rotavirus vaccine, by outcome and age group.

	Mean annual cases		IR /100,000 (95% CI)^a^		IRR (95% CI)	P-value
	Pre-RV vaccine	Post-RV vaccine	Pre-RV vaccine	Post-RV vaccine		
*National all-cause diarrhoea admissions*						
*<1yr*	175.8	107.0	1050 (982, 1121)	615 (558, 676)	0.59 (0.52, 0.66)	<0.001
*1–2yrs*	148.0	84.0	884 (822, 950)	483 (432, 537)	0.55 (0.48, 0.62)	<0.001
*3–4yrs*	158.0	117.0	315 (293, 337)	224 (204, 245)	0.71 (0.63, 0.80)	<0.001
*5–9yrs*	85.6	57.0	108 (98, 119)	69 (61, 79)	0.64 (0.54, 0.75)	<0.001
*10–19yrs*	79.4	47.0	48 (44, 53)	28 (24, 32)	0.57 (0.48, 0.68)	<0.001
*20–54yrs*	194.8	163.0	46 (43, 49)	37 (34, 40)	0.80 (0.73, 0.89)	<0.001
*≥55y*	78.8	95.5	83 (75, 91)	96 (87, 107)	1.17 (1.01, 1.35)	0.017
*Total population*	920.4	670.5	109 (106, 112)	76 (73, 79)	0.70 (0.67, 0.74)	<0.001
*RV diarrhoea admissions at CWMH*						
*≤2m*	1.6	0.5	126 (54, 248)	35 (4, 126)	0.28 (0.03, 1.38)	0.090
*<1yr*	55.6	4.8	731 (647, 821)	70 (45, 103)	0.10 (0.06, 0.14)	<0.001
*1–2yrs*	41.2	5.2	541 (470, 620)	75 (49, 110)	0.14 (0.09, 0.21)	<0.001
*3–4yrs*	23.0	4.0	101 (83, 121)	19 (12, 30)	0.19 (0.11, 0.31)	<0.001
*<5yrs*	119.8	14.0	315 (290, 341)	41 (32, 51)	0.13 (0.10, 0.17)	<0.001
*RV diarrhoea at Savusavu outpatients*						
*<1yr*	2.4	0.0	181 (49, 463)	0 (0, 143)	0.00 (0.00, 1.29)	0.045
*1–2yrs*	2.4	1.5	181 (49, 463)	116 (24, 339)	0.64 (0.09, 3.79)	0.582
*3–4yrs*	1.8	1.0	45 (9, 132)	26 (3, 93)	0.57 (0.05, 4.97)	0.708
*<5yrs*	6.6	2.5	100 (50, 178)	39 (13, 90)	0.39 (0.11, 1.21)	0.077

RV=rotavirus.

IRR= incidence rate ratio.

a Population denominators are included in Supplementary Table 2.

All-cause admissions (the control) increased in all age groups apart from the 10–19 years (where there was no change) and ≤2 months age group where admissions declined. For all the age groups that included infants ≤2 months, namely: ≤2months, <1 year, <5 years and the total population; there were also declines in all-cause admissions (Fig. 1 and Supplementary Table 2).

### 3.3 Vaccine impact on rotavirus positive diarrhoea admissions

Rotavirus diarrhoea admissions at CWMH among children <5 years declined by 87% in the post-rotavirus vaccine period (incidence rate ratio (IRR)=0•13, 95%CI; 0•10–0•17, *p*-value<0•001) (Fig. 1 and Supplementary Table 2). The greatest impact was seen in the <1 year age group (IRR=0•10, 95%CI; 0•06–0•14, *p*-value<0•001) and 1–2 years age group (IRR=0•14, 95%CI; 0•09–0•21, *p*-value<0•001) (Table 3, Fig. 1 and Supplementary Table 2). There was an estimated 72% decline in infants ≤2 months of age (IRR=0•28, 95%CI; 0•03–1•38, *p*-value=0•090) (Fig. 1 and Supplementary Table 2). However, the confidence intervals were wide and spanned one, as there were very few rotavirus cases in this age group (*n* = 10) and therefore considerable uncertainty around this estimate. The results of sensitivity analysis using multiple imputation to adjust for missing stool samples were similar although vaccine impact was slightly less; IRR=0.18 (95%CI; 0.15, 0.24, *p*-value<0•001) among children <5 years (Supplementary Table 2).

**Fig. 2:**
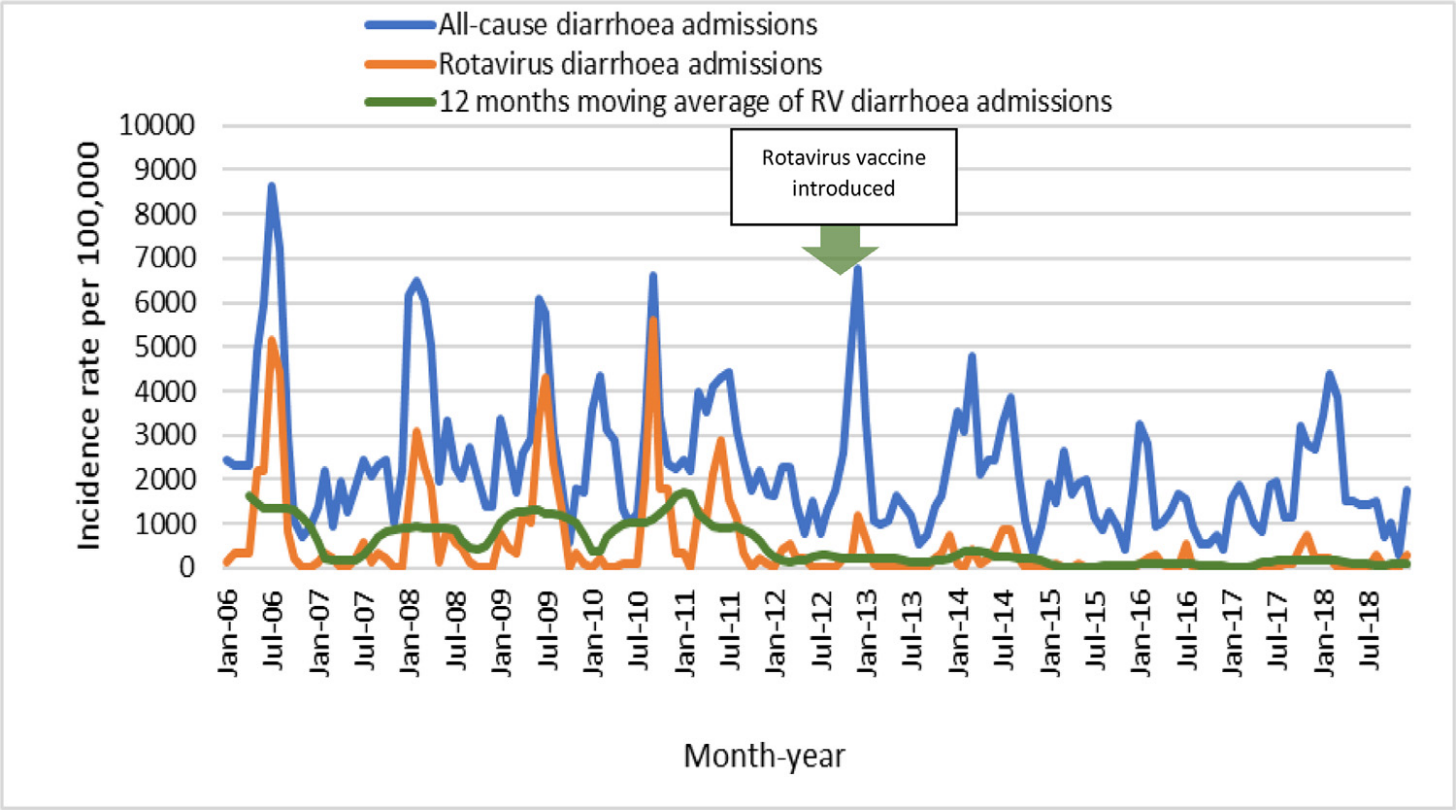
Monthly admissions at Colonial War Memorial Hospital among children <5 years of age for all-cause diarrhoea, rotavirus diarrhoea and the 12 months moving average for rotavirus diarrhoea, between January 2006 and December 2018. The rotavirus vaccine was introduced in October 2012.

**Fig. 3:**
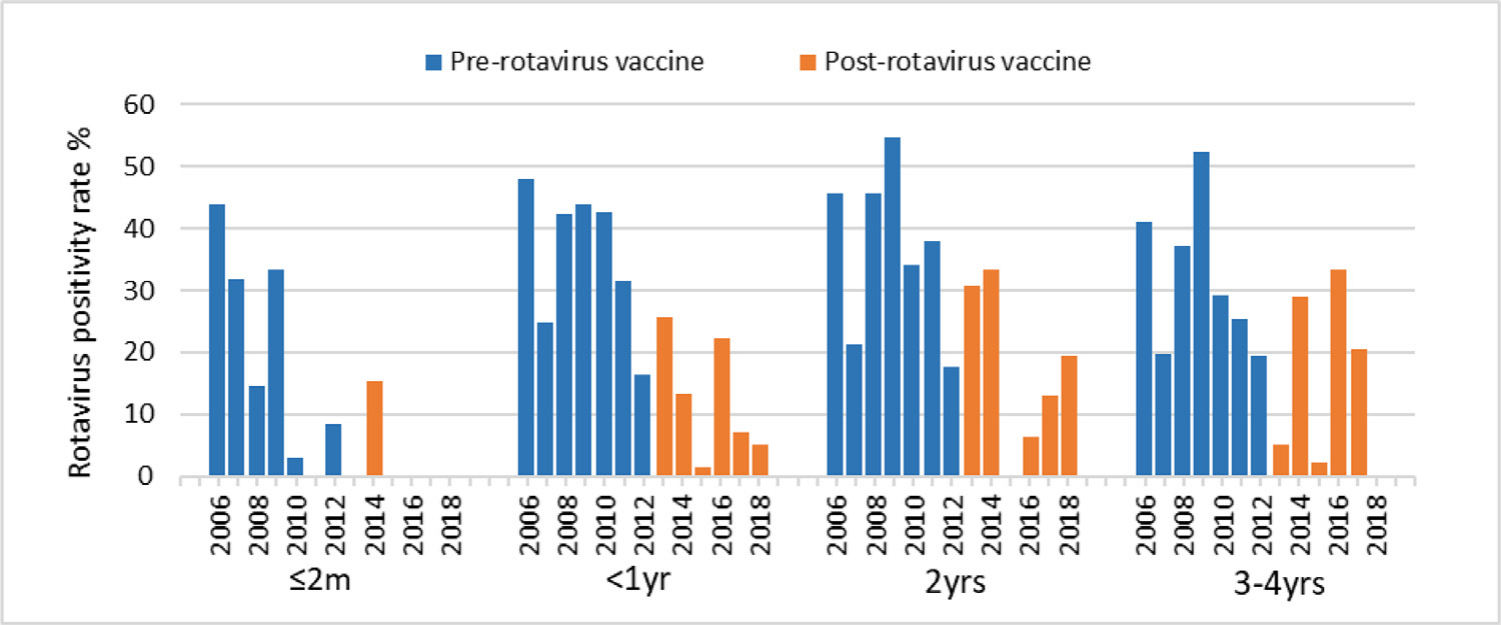
Annual positivity rate for rotavirus diarrhoea admissions at CWMH among children <5 yrs by age group.

Fig. 2 shows the peaks of rotavirus transmission pre-vaccine were blunted in the post-rotavirus vaccine period. Fig. 3 shows the decline in the 3–4 years age group in 2012 and 2013, before this age group were vaccinated.

There was a 81% (95%CI; 51–94%, *p*-value<0•001) decline in the case fatality rate (CFR) for all-cause diarrhoea admissions among children <5 years at CWMH in the pre-rotavirus vaccine period (CFR 2•6%, 95%CI; 1•9–3•5%) vs. in the post-rotavirus vaccine period (CFR 0•5%, 95%CI; 0•2–1•2%).

At Savusavu Hospital, rotavirus diarrhoea outpatient presentations for children <5 years showed an IRR of 0.39 (0.11–1.21, *p*-value=0.077) (Table 3 and Supplementary Table 2). The results of sensitivity analysis using multiple imputation to adjust for missing stool samples were IRR=0.11 (95%CI; 0.04, 0.29, *p*-value<0•001) among children <5 years (Supplementary Table 2). The numbers of cases in this outcome were small (*n* = 15).

The impact of rotavirus vaccine on rotavirus positive diarrhoea among children <5 years was similar among both ethnic groups. There was no evidence for an interaction between vaccine period and ethnicity among children <5 years (*p*-value=0.26); we therefore do not present ethnicity-stratified results.

## 4 Discussion

This study from Fiji found a large reduction in rotavirus confirmed diarrhoea in both hospital admissions and outpatient presentations in children <5 years of age, after the introduction of the rotavirus vaccine nationwide - the first such demonstration from a LMIC in the Western Pacific. There was a reduction in mortality from all-cause diarrhoea admissions in children <5 years. In addition, there were reductions in all-cause diarrhoea admissions in age groups too young or too old to be vaccinated.

There were demonstrable declines in both the severity (81% reduction in mortality), the total burden of rotavirus (>80% decline) and all-cause diarrhoea (36% decline) among children <5 years. These reductions were most likely due to the vaccine as rotavirus diarrhoeal outbreaks remained blunted for the five years after vaccine introduction. Concurrent changes in patient admission policy and management were not observed, evidenced by no decline in all-cause admissions (the control group) except among infants ≤2 months old due to changes in admission criteria for newborns, unrelated to diarrhoea. In Savusavu, outpatient presentations (though small in number) fell, which indicate primary prevention through vaccination, as other interventions such as water, sanitation or hygiene improvements did not occur during the study period. The time series analysis in national all-cause diarrhoea among children <5 years showed a slightly greater decline (52%) to our IRR estimate with little evidence of a change in the background rate of diarrhoea. The >80% reduction in rotavirus diarrhoea admissions among children <5 years of age observed in our study, is greater than that seen in Tanzania (65%) [20], Japan (70%) [21], Armenia (69%) [22] and the Philippines (up to 63%) [11], although similar to Thailand (88%) [23]. This is likely due to the high, full-dose (two doses) vaccine coverage and, perhaps, the lower rates of malnutrition and gut enteropathy than those seen in countries like Botswana where vaccine effectiveness is lower [24].

The greatest reduction in rotavirus diarrhoea and all-cause diarrhoea admissions was among infants <1 year which was sustained into the second year of life. A systematic review of 58 post-licensure studies showed vaccine effectiveness tended to decline in the second year, particularly in medium- and high-mortality settings [25]. Suggested reasons include the placebo group achieving immunity through natural infection, accelerated waning of immunity, and co-infection with a second pathogen [26]. Timing of the vaccine doses may also account for this. While usually given at six and 10 weeks, doses at two weeks and four months (as in Bolivia), led to a measurable (though not quite statistically significant) improvement in vaccine effectiveness [27]. In addition, the highly variable mixing/contact patterns in potentially large households may elicit indirect effects on transmission of rotavirus, thus impacting vaccine effectiveness [28].

This study provides evidence of reductions in diarrhoea among both unvaccinated children and adults in a middle-income setting. Other studies illustrating this are mostly in high-income countries (HIC) including Saudi Arabia [29], the USA [30,31]. In poorer countries this is rare but has been reported, eg. Rwanda [32]. We also found a 70% decline in rotavirus diarrhoea admissions among the infants too young to be vaccinated. While cases were few in Fiji, it is an important public health finding regarding vulnerable infants less than 6 weeks old. Further protection may come with the RV3-BB vaccine, as a phase III clinical trial in Indonesian neonates vaccinated within the first five days of life demonstrated 75% vaccine efficacy against severe rotavirus gastroenteritis to 12 months compared to 51% with the routine infant two dose schedule at eight and 14 weeks [33]. The RV3-BB vaccine will also address the host related genetic resistance in some human populations to P[6] virus.

In Fijian outpatients aged <5 years, rotavirus diagnosis did not decline with the primary analysis using complete case data but did decline with the sensitivity analysis using multiple imputation. There were very few cases, particularly in the post-rotavirus vaccine period and therefore IRR were very sensitive to changes in case numbers. Other countries, including the Philippines [11] and Zimbabwe [34], have demonstrated a reduction in outpatient rotavirus diarrhoea. While, most studies assess rotavirus impact on inpatient presentations, outpatients bear the greatest burden. The rural setting for our outpatient study, Savusavu, had lower pre-vaccination rates of rotavirus diarrhoea than the capital Suva (perhaps due to other, undiagnosed gastrointestinal pathogens) nonetheless the vaccine's impact was apparent.

### 4.1 Limitations

As with all observational studies, causality (reduction of diarrhoea) cannot be ascribed to the introduction of rotavirus vaccine. There were other interventions which may have contributed to our findings - including the introduction of the World Health Organization's (WHO) integrated management for childhood illness (IMCI) in the later years of the study. However, any improvement in case management would not reduce the number of outpatient rotavirus diarrhoea cases as seen in our study. We were not able to assess the dose effect as coverage was high throughout Fiji and there is much population movement. We only included the nation's largest hospital (CWMH) and one (small) secondary hospital, missing several large and many smaller facilities. We only recorded the in-hospital deaths - most paediatric deaths occur in hospital but the adult deaths (for which we did not assess vaccine impact) may have been underestimated. Additionally, stool collection rates varied by vaccine introduction period which may have introduced bias in the period with low collection rates. To address this, we used multiple imputation as a sensitivity analysis, and coupled with the blunting in rotavirus seasonality we believe our findings show supportive evidence that rotavirus vaccine contributed to the reductions in diarrhoea in the post- rotavirus vaccine period. We have removed readmissions within 30 days however we have not adjusted for repeat episodes of hospitalisation in our analysis.

### 4.2 Conclusion

Fiji is the first upper-middle-income country in the Pacific island region to show a decline in the burden of rotavirus diarrhoea, and all-cause diarrhoea admissions in all ages and mortality in children after rotavirus vaccine introduction. While improved outpatient management practices (WHO IMCI) may have contributed to the decline in diarrhoea, the temporal relationship to vaccine introduction is compelling. The reduction in diarrhoea admissions is likely to have resulted in a considerable reduction of the workload for the health system and therefore reduced costs for managing this condition. In addition, in this setting we have found that vaccine protection continues into the second year of life.

## Author contributions

FMR conceived, designed and initiated study and was the key manuscript reviewer; AWJJ and RR drafted the manuscript; RR and CN performed statistical design and analysis; EKM, JEB, BT performed methodology and key manuscript reviews; LT, JK were CWMH leads; ER, RD, LT, JK, ASK and MK coordinated Ministry facilities, KKF, VG and KJ provided logistical advice, intellectual and physical support. KB, AWJJ and RR coordinated study staff especially data collection by FTR and ET; AWJJ, SC, ST, CDK undertook laboratory work. All authors approved the manuscript for submission.

## Data sharing statement

The data belongs to the Government of Fiji. Raw and analysed data is available upon request following permission being granted by the Government of Fiji, via the Ministry of Health and Medical Services Ethical Review Committee.

## Declaration of Interests

CK reports a patent development of the unlicensed ‘RV3 BB’ rotavirus vaccine currently in clinical trials. JB reports grants from World Health Organization, Bill and Melinda Gates Foundation and the Australian National Health and Medical Research Council, Australian Department of Health, GlaxoSmithKline, and CDC Foundation outside of the submitted work for the development of the ‘RV3 BB’ rotavirus vaccine. CN reports grants from Pfizer Inc. outside the submitted work. The authors have nothing other to declare and no competing interests.

## Appendix Supplementary materials

Image, application 1
