# Supplementary material for: The impact of the rotavirus vaccine on diarrhoea, five years following national introduction in Fiji
Source: Lancet Reg Health West Pac. 2020 Nov 25;6:100053. doi: 10.1016/j.lanwpc.2020.100053 (PMC8315333; doi:10.1016/j.lanwpc.2020.100053)
Supplement: Supplementary file 1 [file mmc1.doc]

**Supplementary material**

**Methods**

*Justification for inclusion of data in the pre- and post-rotavirus vaccine introduction period*

The rotavirus vaccine was introduced in October 2012. The years before January 2012 were defined as pre-rotavirus vaccination period. The years 2012-2013 were excluded. The year 2012 was excluded for two reasons; the first, is that full calendar years were used due to the seasonality of rotavirus, with the peak in the first half of the year in Fiji, therefore the inclusion of the months up until September 2012 would bias the pre-vaccine period with higher rates, the second reason being that hospital admission data are incomplete due to technical changes to the PATIS system in 2012 ^1^. The year 2013 was excluded as a transition year to allow a period for high vaccine coverage to be achieved. We used the same time periods for the primary IRR analysis as well as the time series analysis for which exclusion of transition years is a common method ^2-4^. The years after December 2013 were defined as the post-rotavirus vaccination period. The time frames of each dataset differed by site and outcome and are presented in Table 1. The reasons for the differences in available data were; National data were extracted from PATIS which began to be in use in Fiji in 2007. The CWMH surveillance system was established in December 2005 and the first month of data was excluded to allow for the surveillance system to become reliable in a large hospital setting. The Savusavu surveillance system was established in May 2010, the first month of data was not excluded as the surveillance system was simpler in a small secondary hospital, with fewer cases to enrol and, therefore, considered reliable from the start.

*Multiple imputation analysis for missing stool samples*

For children in the diarrhoea surveillance system without a stool sample collected multiple imputation was used to handle missing data for a rotavirus test result. The imputation model contained variables that were part of the primary analysis (i.e. rotavirus result and study period). The following auxiliary variables were included in the imputation model as they were either associated with a rotavirus result or were associated with missing data status: age (in months), days with diarrhoea, vomiting and length of stay, immunisation status, ethnicity and seasonality (June to September vs October to May). As there were missing values in some of the auxiliary variables, these missing values were also imputed. Multiple imputation was implemented using the chained equations method ^5^. This procedure was chosen, as it can be used to impute missing values in multiple variables and the imputation models can be tailored to the variable type ^3^; logistic regression was used to impute binary variables (rotavirus result, vomiting, immunisation status) and linear regression was used impute continuous variables (days with diarrhoea, length of stay). Multiple imputation was implemented in Stata 15.1 using the “mi impute chained command” and 50 imputed datasets were produced. The incidence rate ratios for the vaccine effect were determined using Poisson regression (using “mi estimate: poisson” command). To obtain the incidence rates and 95% confidence intervals for each time period, we used the “mimrgns” command to obtain predictions based on the previously fitted Poisson regression model. Imputation results were combined using Rubin's combining rules.

*Time series analysis of all-cause diarrhoea in children <5 years*

As a sensitivity analysis we performed an interrupted time series regression analysis of all-cause diarrhoea admissions in children <5 years. Monthly admissions from January 2007 to December 2017 were modelled using a negative binomial model to handle over-dispersion (excluding the period from January 2012 to December 2013 to allow for vaccine introduction, in addition there were changes to the PATIS system in early 2012 which resulted in admission data being incomplete in 2012 ^1^) The model included the following variables: time elapsed since the start of the study (representing the secular trend), a binary variable for vaccine period (represent the level change following vaccine introduction) and indicator variables for calendar month to handle seasonality. The logarithm of monthly population values was included in the model as an offset to enable estimation of incidence rates. The fit of the model was assessed by checking residuals.

**Results**

*Time series analysis of all-cause diarrhoea in children <5 years*

Results of the times series analysis are shown in Supplementary Table 3 and Supplementary Figure 1. The level change of 0.477 indicates that there was a 52.3% (95% CI: 27.7% to 68.5%) decrease in monthly all-cause diarrhoea admissions following vaccine introduction. Supplementary Figure 1 shows a time series plot for all-cause diarrhoea admissions, 2007-2012. Results of the times series analysis are shown in Supplementary Figure 1.

**Supplementary Table 1. Demographics and clinical characteristics of non-bloody diarrhoea admissions in children <5yrs of age admitted to CWMH and Savusavu Hospital between 2007 to 2017, in children with and without stool collected**

| **Characteristics** | **CWMH all-cause diarrhoea admissions** | | | | **Savusavu all-cause diarrhoea admissions** | | | | **Savusavu all-cause diarrhoea outpatient presentations** | | | |
| --- | --- | --- | --- | --- | --- | --- | --- | --- | --- | --- | --- | --- |
|  | **Total n=3,312** | **Stool specimen tested**  **n=2,314** | **No stool specimen tested n=998** | **P-value** | **Total**  **n=179** | **Stool specimen tested**  **n=127** | **No stool specimen tested**  **n=52** | **P-value** | **Total n=1,260** | **Stool specimen tested**  **n=761** | **No stool specimen tested**  **n=499** | **P-value** |
| **Age in months, median (IQR)** | n=3,311 | n=2,313 | n=998 |  | n=179 | n=127 | n=52 |  | n=1,259 | n=760 | n=499 |  |
|  | 13 (7-23) | 12 (7-23) | 14 (8-24) | 0.06 | 17 (11-29) | 18 (12-28) | 15 (8-32) | 0.29 | 19 (11-30) | 20 (12-30) | 18 (11-30) | 0.01 |
| **Ethnicity, n (%)** | n=2,982 | n=2,169 | n=813 |  | n=89 | n=46 | n=43 |  | n=498 | n=161 | n=337 |  |
| i-Taukei | 2,347 (78.7) | 1,695 (78.1) | 652 (80.2) |  | 79 (88.8) | 42 (91.3) | 37 (86.1) |  | 335 (67.3) | 118 (73.3) | 217 (64.4) |  |
| Fijians of Indian descent | 447 (15.0) | 331 (15.3) | 116 (14.3) |  | 5 (5.6) | 1 (2.2) | 4 (9.3) |  | 117 (23.5) | 32 (19.9) | 85 (25.2) |  |
| Others | 188 (6.3) | 143 (6.6) | 45 (6.6) | 0.41 | 5 (5.6) | 3 (6.5) | 2 (4.7) | 0.33 | 46 (9.2) | 11 (6.8) | 35 (10.4) | 0.13 |
| **Male, n (%)** | n=3,311 | n=2,313 | n=998 |  | n=179 | n=127 | n=52 |  | n=1,260 | n=761 | n=499 |  |
|  | 1,967 (59.4) | 1,362 (58.9) | 605 (60.7) | 0.35 | 101 (56.4) | 70 (55.1) | 31 (59.6) | 0.58 | 714 (56.7) | 445 (58.5) | 269 (53.9) | 0.11 |
| **Length of stay in days, median (IQR)** | n=3,180 | n=2,110 | n=869 |  | n=75 | n=36 | n=39 |  |  |  |  |  |
|  | 2 (1-4) | 2 (1-40) | 2 (1-4) | <0.001 | 3 (1-4) | 3 (2-5) | 3 (1-4) | 0.09 | - | - | - | - |
| **Days with diarrhoea before admission, median (IQR)** | n=2,583 | n=1,862 | n=721 |  | n=76 | n=38 | n=38 |  | n=180 | n=45 | n=135 |  |
|  | 2 (1-3) | 2 (1-3) | 2 (1-3) | 0.99 | 2 (1-4) | 2 (1-4) | 2 (1-4) | 0.50 | 2 (1-3) | 2 (1-3) | 2 (1-1) | 0.12 |
| **Received IV fluid, n (%)** | n=2,685 | n=1,904 | n=781 |  | n=161 | n=121 | n=49 |  | n=1,048 | n=661 | n=381 |  |
|  | 1,663 (61.9) | 1,246 (65.4) | 417 (53.4) | <0.001 | 94 (58.4) | 73 (65.2) | 21 (42.9) |  | 16 (1.5) | 6 (1.6) | 10 (1.5) | 0.96 |
| **Vomiting, n (%)** | n=1,425 | n=1,206 | n=219 |  | n=81 | n=40 | n=41 |  | n=349 | n=98 | n=251 |  |
|  | 1,127 (79.1) | 956 (79.3) | 171 (78.1) | 0.69 | 52 (64.2) | 29 (72.5) | 23 (56.1) | 0.12 | 156 (44.7) | 33 (33.7) | 123 (49.0) | 0.01 |
| **Died during admission, n (%)** | n=3,206 | n=2,234 | n=972 |  | n=174 | n=123 | n=51 |  | n=1,225 | n=738 | n=487 |  |
|  | 61 (1.9) | 36 (1.6) | 25 (2.6) | 0.07 | 0 | 0 | 0 | - | 3 (<1%) | 2 (<1%) | 1 (<1%) | 0.82 |
| **Rotavirus vaccination period, n (row %)** | n=2,891¹ | n=2,084 | n=807 |  | n=123 | n=82 (66.7) | n=41 |  | n=867 | n=511 | n=356 |  |
| Pre-rotavirus vaccination period | 1854 (100) | 1,498 (80.8) | 356 (19.2) | <0.001 | 84 (100.0) | 44 (52.4) | 40 (47.6) | <0.001 | 347 (100.0) | 79 (22.8) | 268 (77.2) | <0.001 |
| Post-rotavirus vaccination period | 1037 (100) | 586 (56.5) | 451 (43.5) |  | 39 (100.0) | 39 (97.44) | 1 (2.6) |  | 520 (100.00) | 432 (83.1) | 88 (16.9) |  |

¹Data are not presented for cases occurring between 2012-2013 which were excluded from the vaccine impact analysis. The number of cases not presented by facility are; CWMH inpatients n=421, Savusavu inpatients n=56 and Savusavu outpatients n=392.

**Supplementary Table 2. Mean annual cases and incidence rates in the pre- and post-rotavirus vaccine periods in Fiji, and incidence rate ratios estimating post/pre-rotavirus vaccine, by outcome and age group**

|  | **Mean annual population** | | **Mean annual cases** | | **IR /100,000 (95% CI)** | | **IRR (95% CI)** | **P-value** |
| --- | --- | --- | --- | --- | --- | --- | --- | --- |
|  | **Pre-RV vaccine** | **Post-RV vaccine** | **Pre-RV vaccine** | **Post-RV vaccine** | **Pre-RV vaccine** | **Post-RV vaccine** |  |  |
| **National all-cause diarrhoea admissions** | | | | | | | |  |
| **≤2m** | 2790.6 | 2901.3 | 8.2 | 12.8 | 294 (211, 398) | 439 (327, 577) | 1.50 (0.97, 2.31) | 0.0434 |
| **1-11ms** | 15348.4 | 15956.5 | 171.4 | 102.5 | 1117 (1044, 1194) | 642 (582, 707) | 0.58 (0.51, 0.65) | <0.001 |
| **<1yr** | 16743.8 | 17407.3 | 175.8 | 107.0 | 1050 (982, 1121) | 615 (558, 676) | 0.59 (0.52, 0.66) | <0.001 |
| **1-2yrs** | 16743.8 | 17407.3 | 148.0 | 84.0 | 884 (822, 950) | 483 (432, 537) | 0.55 (0.48, 0.62) | <0.001 |
| **3-4yrs** | 50230.2 | 52221.0 | 158.0 | 117.0 | 315 (293, 337) | 224 (204, 245) | 0.71 (0.63, 0.80) | <0.001 |
| **<5yrs** | 83717.8 | 87035.5 | 481.8 | 308.0 | 576 (553, 599) | 354 (334, 374) | 0.61 (0.57, 0.66) | <0.001 |
| **5-9yrs** | 78960.8 | 82090.8 | 85.6 | 57.0 | 108 (98, 119) | 69 (61, 79) | 0.64 (0.54, 0.75) | <0.001 |
| **10-19yrs** | 163856.6 | 170351.0 | 79.4 | 47.0 | 48 (44, 53) | 28 (24, 32) | 0.57 (0.48, 0.68) | <0.001 |
| **20-54yrs** | 425607.8 | 442476.8 | 194.8 | 163.0 | 46 (43, 49) | 37 (34, 40) | 0.80 (0.73, 0.89) | <0.001 |
| **≥55y** | 95237.2 | 99011.8 | 78.8 | 95.5 | 83 (75, 91) | 96 (87, 107) | 1.17 (1.01, 1.35) | 0.017 |
| **Total population** | 847380.2 | 880965.8 | 920.4 | 670.5 | 109 (106, 112) | 76 (73, 79) | 0.70 (0.67, 0.74) | <0.001 |
| **RV diarrhoea admissions at CWMH** | | | | | | | |  |
| **≤2m** | 1268.6 | 1150.2 | 1.6 | 0.5 | 126 (54, 248) | 35 (4, 126) | 0.28 (0.03, 1.38) | 0.045 |
| **<1yr** | 7610.4 | 6900.8 | 55.6 | 4.8 | 731 (647, 821) | 70 (45, 103) | 0.10 (0.06, 0.14) | <0.001 |
| **1-2yrs** | 7610.4 | 6900.8 | 41.2 | 5.2 | 541 (470, 620) | 75 (49, 110) | 0.14 (0.09, 0.21) | <0.001 |
| **3-4yrs** | 22833.6 | 20704.0 | 23.0 | 4.0 | 101 (83, 121) | 19 (12, 30) | 0.19 (0.11, 0.31) | <0.001 |
| **<5yrs** | 38054.4 | 34505.6 | 119.8 | 14.0 | 315 (290, 341) | 41 (32, 51) | 0.13 (0.10, 0.17) | <0.001 |
| **RV diarrhoea admissions at CWMH using imputed data, results averaged over 50 imputations** | | | | | | | | |
| **≤2m** | 1268.6 | 1150.2 | 2.5 | 0.8 | 195 (85, 309) | 71 (-11, 152) | 0.34 (0.89, 1.37) | 0.072 |
| **<1yr** | 7610.4 | 6900.8 | 66.0 | 9.8 | 868 (769, 966) | 142 (92, 192) | 0.16 (0.11, 0.23) | <0.001 |
| **1-2yrs** | 7610.4 | 6900.8 | 50.2 | 8.1 | 659 (572, 746) | 117 (73, 160) | 0.18 (0.12, 0.26) | <0.001 |
| **3-4yrs** | 22833.6 | 20704.0 | 29.1 | 6.6 | 127 (105, 150) | 32 (19, 44) | 0.25 (0.16, 0.38) | <0.001 |
| **<5yrs** | 38054.4 | 34505.6 | 145.3 | 24.5 | 382 (352, 412) | 72 (55, 87) | 0.18 (0.15, 0.24) | <0.001 |
| **RV diarrhoea at Savusavu inpatients** | | | | | | | | |
| **<1yr** | 1326.1 | 1293.0 | 0.6 | 0.0 | 45 (1, 252) | 0 (0, 143) | 0.0 (0.0, 33.33) | 0.4608 |
| **1-2yrs** | 1326.1 | 1293.0 | 3.0 | 0.0 | 226 (74, 527) | 0 (0, 143) | 0.0 (0.0, 0.93) | 0.0208 |
| **3-4yrs** | 3977.6 | 3878.5 | 2.4 | 0.0 | 60 (16, 154) | 0 (0, 48) | 0.0 (0.0, 1.29) | 0.0657 |
| **<5yrs** | 6629.7 | 6464.5 | 6.0 | 0.0 | 91 (43, 166) | 0 (0, 29) | 0.0 (0.0, 0.38) | 0.0004 |
| **RV diarrhoea at Savusavu inpatients  using imputed data, results averaged over 50 imputations** | | | | | | | | |
| **<1yr** | 1326.1 | 1293.0 | 2.9 | 0.0 | 266 (-18, 550) | 0 (-0.01, 0.01) | 0.0 (0.0, 0.0) | 0.998 |
| **1-2yrs** | 1326.1 | 1293.0 | 4.6 | 0.0 | 419 (101, 737) | 0 (-0.01, 0.01) | 0.0 (0.0, 0.0) | 0.998 |
| **3-4yrs** | 3977.6 | 3878.5 | 3.1 | 0.1 | 93 (4, 181) | 1 (-10, 12) | 0.0 (0.0, 0.0) | 0.998 |
| **<5yrs** | 6629.7 | 6464.5 | 10.6 | 0.1 | 193 (82, 303) | 1 (-6, 7) | 0.0 (0.0, 0.0) | 0.997 |
| **RV diarrhoea at Savusavu outpatients** | | | | | | | | |
| **<1yr** | 1326.1 | 1293.0 | 2.4 | 0.0 | 181 (49, 463) | 0 (0, 143) | 0.0 (0.0, 1.29) | 0.09 |
| **1-2yrs** | 1326.1 | 1293.0 | 2.4 | 1.5 | 181 (49, 463) | 116 (24, 339) | 0.64 (0.09, 3.79) | 0.582 |
| **3-5yrs** | 3977.6 | 3878.5 | 1.8 | 1.0 | 45 (9, 132) | 26 (3, 93) | 0.57 (0.05, 4.97) | 0.708 |
| **<5yrs** | 6629.7 | 6464.5 | 6.6 | 2.5 | 100 (50, 178) | 39 (13, 90) | 0.39 (0.11, 1.21) | 0.077 |
| **RV diarrhoea at Savusavu outpatients using imputed data, results averaged over 50 imputations** | | | | | | | | |
| **<1yr** | 1326.1 | 1293.0 | 8.1 | 0.4 | 733 (166, 1300) | 32 (-66, 129) | 0.0 (0.0, 0.0) | 0.998 |
| **1-2yrs** | 1326.1 | 1293.0 | 11.5 | 1.9 | 1042 (341, 1743) | 143 (-19, 305) | 0.14 (0.04, 0.50) | 0.004 |
| **3-4yrs** | 3977.6 | 3878.5 | 9.6 | 1.3 | 288 (38, 539) | 35 (-15, 85) | 0.12 (0.03, 0.59) | 0.009 |
| **<5yrs** | 6629.7 | 6464.5 | 29.2 | 3.6 | 528 (226, 830) | 56 (4, 107) | 0.11 (0.04, 0.29) | <0.001 |
| **National all-cause admissions excluding pneumonia, diarrhoea, obstetric and mental health admissions** | | | | | | | | |
| **≤2m** | 845984.8 | 879515.0 | 4040.2 | 1605.8 | 478 (471, 484) | 183 (178, 187) | 0.38 (0.37, 0.39) | <0.001 |
| **1-11ms** | 15348.4 | 15956.5 | 545.6 | 732.8 | 3555 (3425, 3688) | 4592 (4431, 4757) | 1.29 (1.23, 1.36) | <0.001 |
| **<1yr** | 16743.8 | 17407.3 | 4477.2 | 2182.0 | 26739 (26440, 27041) | 12535 (12290, 12783) | 0.47 (0.46, 0.48) | <0.001 |
| **1-2yrs** | 16743.8 | 17407.3 | 423.6 | 581.8 | 2530 (2425, 2639) | 3342 (3210, 3478) | 1.32 (1.24, 1.40) | <0.001 |
| **3-4yrs** | 50230.2 | 52221.0 | 722.8 | 974.3 | 1439 (1393, 1486) | 1866 (1808, 1925) | 1.30 (1.24, 1.36) | <0.001 |
| **1m-5yrs** | 80878.8 | 84084.0 | 4040.2 | 2288.8 | 2092 (2048, 2137) | 2722 (2667, 2778) | 1.30 (1.26, 1.34) | <0.001 |
| **<5yrs** | 83717.8 | 87035.5 | 5623.6 | 3738.0 | 6717 (6642, 6794) | 4295 (4228, 4363) | 0.64 (0.63, 0.65) | <0.001 |
| **5-9yrs** | 78960.8 | 82090.8 | 768.0 | 1039.5 | 973 (942, 1004) | 1266 (1228, 1305) | 1.30 (1.25, 1.36) | <0.001 |
| **10-19yrs** | 163856.6 | 170351.0 | 1623.6 | 1709.3 | 991 (970, 1013) | 1003 (980, 1027) | 1.01 (0.98, 1.05) | 0.5773 |
| **20-54yrs** | 425607.8 | 442476.8 | 8911.2 | 9464.8 | 2094 (2075, 2113) | 2139 (2118, 2160) | 1.02 (1.01, 1.04) | 0.0013 |
| **≥55y** | 95237.2 | 99011.8 | 5500.2 | 6709.5 | 5775 (5709, 5842) | 6776 (6698, 6855) | 1.17 (1.15, 1.19) | <0.001 |
| **Total excluding neonates** | 845984.8 | 703612.0 | 18495.0 | 21211.8 | 2186 (2172, 2200) | 2412 (2396, 2428) | 1.10 (1.09, 1.11) | <0.001 |
| **Total** | 847380.2 | 880965.8 | 22426.6 | 22661.0 | 2647 (2631, 2662) | 2572 (2556, 2589) | 0.97 (0.96, 0.98) | <0.001 |

RV=rotavirus, IRR= incidence rate ratio

^1^ The upper limit could not be estimated because there were zero cases in the post-vaccine period.

**Supplementary Table 3. Results of sensitivity analysis – time series regression with level change. Results shown are incidence rate ratios (IRR), 95% confidence intervals and p-values for the negative binomial model fitted to monthly all-cause diarrhoea admissions in children <5 years between 2007-2017.**

|  | **Incidence rate ratio**  **(95%confidence interval)** | **P-value** |
| --- | --- | --- |
| **Level change after vaccine introduction** | 0.48 (0.31, 0.72) | <0.001 |
| **Time since study commencement (months)** | 1.00 (1.00, 1.01) | 0.21 |
| **Calendar month** |  |  |
| January | Reference | - |
| February | 1.00 (0.69, 1.45) | 0.98 |
| March | 1.01 (0.69, 1.46) | 0.98 |
| April | 0.93 (0.64, 1.35) | 0.69 |
| May | 0.79 (0.54, 1.15) | 0.22 |
| June | 0.99 (0.68, 1.44) | 0.96 |
| July | 0.85 (0.58, 1.24) | 0.41 |
| August | 0.85 (0.58, 1.24) | 0.40 |
| September | 0.73 (0.50, 1.06) | 0.10 |
| October | 0.64 (0.44, 0.94) | 0.02 |
| November | 0.54 (0.37, 0.80) | 0.00 |
| December | 0.70 (0.48, 1.03) | 0.07 |

**Figure 2. Time series plot for monthly all-cause diarrhoea admissions, 2007-2012. The plot shows observed values (blue line), fitted values from the model (red line), and model-based counterfactual values (dashed line) that are predicted to occur in the absence of vaccine introduction. The interval between the vertical dotted lines is the vaccine introduction period. The unit for the x-axis is month.**

**References**

1. Reyburn R, Nand D, Nguyen C, et al. Validation of administrative data to estimate vaccine impact: Audit of the Fiji hospital admissions electronic database, 2007-2011 & 2014-2015. *Vaccine* 2017; **35**(47): 6416-21.

2. Baker JM, Dahl RM, Cubilo J, Parashar UD, Lopman BA. Effects of the rotavirus vaccine program across age groups in the United States: analysis of national claims data, 2001-2016. *BMC Infect Dis* 2019; **19**(1): 186.

3. Silaba M, Ooko M, Bottomley C, et al. Effect of 10-valent pneumococcal conjugate vaccine on the incidence of radiologically-confirmed pneumonia and clinically-defined pneumonia in Kenyan children: an interrupted time-series analysis. *Lancet Glob Health* 2019; **7**(3): e337-e46.

4. DeAntonio R, Amador S, Bunge EM, et al. Vaccination herd effect experience in Latin America: a systematic literature review. *Hum Vaccin Immunother* 2019; **15**(1): 49-71.

5. White IR, Royston P, Wood AM. Multiple imputation using chained equations: Issues and guidance for practice. *Stat Med* 2011; **30**(4): 377-99.
